# Seasonal and stable heterotrophic guilds drive Arctic benthic microbiome functioning across polar day and night

**DOI:** 10.1093/ismeco/ycaf161

**Published:** 2025-09-19

**Authors:** Chyrene Moncada, Carol Arnosti, Jan D Brüwer, Dirk de Beer, Gunter Wegener, Peter Stief, Marit R van Erk, Jürgen Titschack, Rudolf Amann, Katrin Knittel

**Affiliations:** Max Planck Institute for Marine Microbiology, 28359 Bremen, Germany; Department of Earth, Marine, and Environmental Sciences, University of North Carolina at Chapel Hill, Chapel Hill, NC 27599, United States; Max Planck Institute for Marine Microbiology, 28359 Bremen, Germany; Max Planck Institute for Marine Microbiology, 28359 Bremen, Germany; Max Planck Institute for Marine Microbiology, 28359 Bremen, Germany; MARUM, Center for Marine Environmental Sciences, University of Bremen, 28359, Bremen, Germany; Max Planck Institute for Marine Microbiology, 28359 Bremen, Germany; HADAL & Nordcee, Department of Biology, University of Southern Denmark, 5230 Odense M, Denmark; Max Planck Institute for Marine Microbiology, 28359 Bremen, Germany; Department of Microbiology, Radboud Institute for Biological and Environmental Sciences, Radboud University, 6525 AJ Nijmegen, The Netherlands; MARUM, Center for Marine Environmental Sciences, University of Bremen, 28359, Bremen, Germany; Marine Research Department, Senckenberg am Meer, 26382 Wilhelmshaven, Germany; Max Planck Institute for Marine Microbiology, 28359 Bremen, Germany; Max Planck Institute for Marine Microbiology, 28359 Bremen, Germany

**Keywords:** sandy sediments, porewater, microbial community, fraction of dividing cells, hydrolysis rates, oxygen consumption, microbial diversity

## Abstract

The remineralization of organic matter by benthic bacteria is an essential process in the marine carbon cycle. In polar regions, strong variation in daylength causes pronounced seasonality in primary productivity, but the responses of sedimentary bacteria to these fluctuations are not well understood. We investigated the seasonal dynamics of benthic bacterial communities from an Arctic fjord and found a partitioning of the communities into seasonally responsive and stable guilds. We separately analyzed the fractions of cells in the porewater and those loosely and firmly attached to sand grains through 16S ribosomal RNA gene sequencing, cell counting, rate measurements, and geochemical analyses. The porewater and loosely attached bacterial communities showed a dynamic response in composition and activity, suggesting that they play a central role in benthic–pelagic coupling by responding rapidly to seasonal fluctuations in organic matter availability. In contrast, the majority of the firmly attached cells showed a more buffered response, as reflected, e.g. in the consistently high cell numbers of *Woeseiaceae*. This fraction is potentially key to maintaining baseline remineralization processes throughout the year, independent of fresh organic matter input. These findings provide a new mechanistic understanding of carbon cycling in Arctic surface sediments that may also apply beyond polar regions.

## Introduction

High-latitude marine environments are characterized by extreme seasonal changes, with extended periods of darkness (polar night) followed by a rapid transition to months of continuous sunlight (polar day). This variation leads to strong shifts in the phototrophic and heterotrophic microbial communities, as has been shown in the water column [1–4]. The return of light initiates a phytoplankton bloom that is typically diatom-dominated [5, 6] and accounts for much of the annual net primary production in Arctic marine ecosystems [7, 8]. The polar night begins when solar irradiance declines, and photosynthetic primary production halts [9]. In addition to pronounced seasonal fluctuations in primary productivity, ongoing perturbations such as rising sea surface temperatures and enhanced inflow of Atlantic water, as well as greater discharge of terrestrial material into the Arctic Ocean, are expected to further alter the timing and magnitude of substrate input [10–14].

Understanding how microbial communities in Arctic environments respond to such variability is essential to assess how these communities can buffer or amplify the effects of climate-induced changes in organic matter supply. Most of the knowledge of seasonal microbial community dynamics in the Arctic, however, derives from studies of the water column [1, 3, 15, 16]. Although many studies on benthic communities in the Arctic have shown a highly active microbial community that is not limited by low temperatures but rather by substrate availability (for a review, see [17]), few investigations to date have focused on the response of benthic bacteria to the extreme variability in organic matter supply [2, 18, 19]. A survey of bacterial communities in sandy sediments from Isfjorden, Svalbard, found a stable composition in bulk surface sediments across seasons [2]. A seasonal response was only observed at the gene expression level, in particular in the expression of some carbohydrate-active enzymes [18]. However, bulk characterization of benthic bacterial communities fails to distinguish between the distinct microhabitats within sediments—such as grain surfaces and porewater—thereby potentially masking important differences within the community. In other environments such as freshwater lakes and Middle Atlantic Bight shelf sands, the porewater communities showed limited overlap with the surrounding communities, sharing only 12%–19% of operational taxonomic units with sediments in lakes and 2%–3% with the sediment and seawater in shelf sands [20, 21]. Bacteria in these microhabitats may experience differences in access to particulate and dissolved organic matter, as well as to oxygen and other electron acceptors. This microscale heterogeneity, which is not assessed in bulk sediment investigations, influences the activity, physiology, and interactions of microorganisms, which ultimately impacts biogeochemical cycles at larger scales [22–24].

Recently, we found striking contrasts in the composition and activity of the bacterial communities across microhabitats in sandy surface sediments in Isfjorden (Svalbard) [25]. We disentangled the benthic microbial community using a novel fractionation method that separates cells into three distinct fractions: cells in the porewater (PW), cells loosely attached (LA) to sediment grains, and cells firmly attached (FA) to grains. The PW and LA fractions were enriched in aerobic heterotrophs, potentially responding to fresh input of high-molecular-weight organic matter. In contrast, the less active FA fraction was enriched in anaerobes and taxa potentially degrading detrital organic matter and smaller molecules [25]. We proposed that the PW and LA fractions occupy microenvironments with stronger advective flow, providing enhanced substrate supply, whereas the FA bacteria may be occupying more diffusion-limited areas such as deep cracks and fissures on the grain [25]. The observed differences among these fractions raise the question: how do they respond to the extreme seasonal variability in primary productivity in the Arctic? To answer this question, we revisited the coastal sampling site in Isfjorden during polar night, polar day spring, and polar day summer. We hypothesize that the PW and LA fractions comprise the seasonally responsive fractions in terms of composition and activity. In contrast, if cells in the FA fraction indeed rely on consistently available substrates such as detrital material or small organic molecules, then seasonal changes in this fraction may be less pronounced. Overall, our results point toward a division of labor (i.e. microbial guilds) within surface sediment communities, which drive seasonal and sustained organic matter remineralization processes. This partitioning likely plays a crucial role in maintaining ecosystem function, particularly in regions with extreme seasonal variability, where fluctuating organic matter availability can significantly impact microbial community dynamics.

## Materials and methods

### Site description and sample collection

Between 2021 and 2023, we seasonally collected surface sediments, overlying seawater (OSW), and surface seawater (SSW) from Isfjorden, Svalbard, at an average depth of 4.5 m (Fig. S1, “Station 5”: 78°06′24.3″N, 14°21′04.20″E ± 30 m). The sampling was done on two separate days per sampling campaign, during polar night (24 h darkness: 8 and 11 December 2021, 20 and 21 January 2023), polar day spring (24 h daylight: 30 April and 2 May 2022, and 27 and 29 April 2023), and polar day summer (29 June and 2 July 2022). SSW samples were obtained using a bucket. Sediments and the OSW were sampled using the Ellrott grab [26]. Across sampling dates, the sediments were comprised mostly of fine sand, and the grain size distribution did not vary across seasons (Fig. S2). Water depths, seawater, and sediment temperatures, salinity, sediment porosity, and oxygen penetration depths are given in Table S1.

The top 2 cm of sediment was subsampled for further fractionation and analyses. Samples for pigment analysis, and carbon and nitrogen measurements were frozen on dry ice on board until analysis; samples to measure dissolved inorganic carbon content of seawater and porewater were stored at +4°C. Quantification of these parameters is detailed in the supplementary materials and methods.

### Fractionation of sediments

The sedimentary microbial community was fractionated into cells in the PW, LA, and FA as described previously [25]. Briefly, to extract the PW, 30 ml of surface sediment was transferred to a 50 ml tube, 5 ml of sterile artificial seawater (ASW: 450 mM NaCl, 59.6 mM MgCl_2_, 56.5 mM MgSO_4_, 13.2 mM CaCl_2_, 9.7 mM KCl, 0.8 mM KBr, 0.3 mM H_3_BO_3_, 0.1 mM SrCl_2_) was added, and the tube was affixed to a 60 μm pore size Steriflip (Merck KgaA, Darmstadt, Germany). The tube was then inverted, and the PW was retrieved by applying a vacuum. To obtain the LA fraction, the porewater-free sediment was shaken in ASW at 1000 rpm for 40 s (BioShake iQ shaker, Q Instruments GmbH, Jena, Germany) to simulate sediment reworking. After six rounds of shaking, the supernatants (i.e. the loosely attached cells) were pooled, and the remaining cells were classified as the FA fraction.

### DNA extraction, 16S rRNA gene sequencing, and analysis

We filtered the SSW and OSW, as well as the PW and LA fractions, through a 0.22 μm pore size Sterivex filter (Merck KgaA, Darmstadt, Germany) and used the membrane for DNA extraction (as in [27]). SSW, OSW, and bulk sediment were frozen directly in dry ice on board, while fractions were frozen after fractionation. The primers S-D-Bact-0341-b-S-17 and S-D-Bact-0785-a-A-21 [28] were used to amplify the V3–V4 region of the 16S rRNA gene. The amplicons were sequenced on a MiSeq (2x300 bp; Illumina Inc., CA, USA) at the Max Planck Genome Center in Cologne, Germany. For the January and April 2023 amplicons, additional sequences were obtained via a NextSeq (2x300 bp; Illumina Inc., CA, USA). Information on the extraction kits used, the amplicon library preparation, and amplicon sequence variant (ASV) analyses are detailed in the supplementary materials and methods.

### Total cell counts, CARD-FISH, and measurement of the fraction of dividing cells

To quantify total cell numbers, samples were fixed with formaldehyde (1.5% final concentration) immediately after retrieval and incubated for 1–4 h at room temperature. Details on the sample processing after fixation are provided in the supplementary materials and methods. Cells were manually counted using a Nikon 50i epifluorescence microscope (Nikon Instruments, Inc., USA).

To identify and quantify major taxa in Svalbard surface sediments, catalyzed reporter deposition-fluorescence *in situ* hybridization (CARD-FISH) was performed on all fractions from December 2021, April 2022, and June 2022. Horseradish peroxidase-labeled probes (biomers.net GmbH, Ulm, Germany) were used following a previously described protocol [29], with modifications described previously [2]. Probe and formamide concentrations are listed in Table S2. Automated imaging of FISH-stained cells was done using a motorized epifluorescence microscope (AxioImager Z2m, Carl Zeiss, Jena, Germany) and subsequent analysis was performed with the ACMEtool 3.0 [30] as previously described [2]. The fraction of dividing cells for *Bacteria*, *Bacteroidota*, *Gammaproteobacteria*, and *Woeseiaceae* in the fractions across seasons was determined by identifying dividing cells based on two intracellular DNA-stain maxima, compared to one in non-dividing cells [31].

### Laminarin extracellular hydrolysis rate measurements

To compare seasonal changes in extracellular laminarin hydrolysis rates within the same sediment fraction (as a proxy for the hydrolysis of photosynthetically derived organic matter), we set up batch incubations with fluorescently-labelled laminarin (FLA-laminarin) for the OSW, PW, LA, FA, and unfractionated bulk sediment. The FLA-laminarin used was synthesized and characterized as described in [32]. The incubation set-ups and timepoints are summarized in Table S3. The incubations were conducted in triplicate at 4°C in the dark, alongside autoclaved killed controls. Subsamples were taken throughout the incubations by collecting 1 ml of supernatant and filtering it through a 0.2 μm filter (SFCA membrane, Thermo Fisher Scientific, MA, USA). Until analysis, the filtered supernatants were frozen at −20°C. Hydrolysis rates were determined by measuring the changes in the molecular weight of FLA-laminarin over time [32] and the rates were calculated as described previously [25].

### Oxygen consumption measurements

In June 2022, January 2023, and April 2023, the rates of oxygen consumption in the fractions were measured using the procedures as previously described [25]. In brief, sediments from at least three grabs were fractionated. Oxygen consumption was determined immediately after fractionation using an oxygen optode (OXF50-OI, Pyroscience GmbH, Aachen, Germany). Oxygen consumption rates were normalized to per-cell rates using the corresponding DAPI cell counts from each sample. These rates reflect total oxygen consumption, encompassing both respiration of organic carbon and the reoxidation of reduced species produced by anaerobic respiration, the sum of which represents the total organic matter degradation [33]. The use of killed controls (e.g. via respiratory inhibitors) was not feasible under our field conditions due to safety and environmental constraints.

## RESULTS

### Sediment pigment content varied seasonally while sediment TOC and TON remained constant

**Figure 1:**
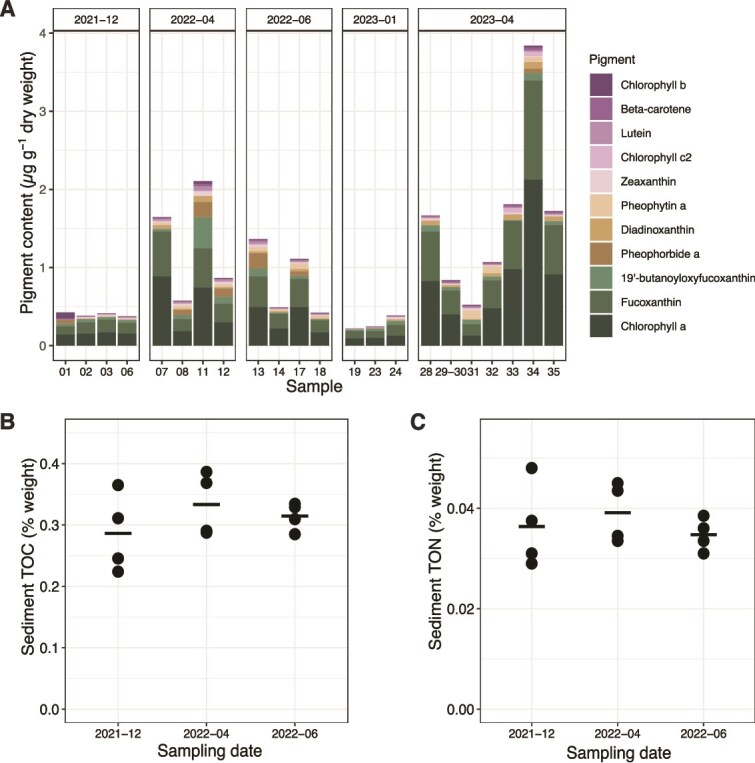
Bulk sediment parameters across seasons. (A) Pigment content (in μg g^−1^ sediment dry weight). Each bar represents one replicate sediment grab. *X*-axis labels indicate the sediment grab number. (B) Total organic carbon (TOC, in % dry weight). (C) Total organic nitrogen content (TON, in % dry weight). Each dot represents one replicate sediment grab. For (B) and (C), the solid horizontal bar represents the mean.

Across seasons, chlorophyll *a* and fucoxanthin were the two most abundant pigments that could be extracted from the bulk sediment (Fig. 1A), with concentrations in the typical range of coastal marine sediments [34]. The fucoxanthin-to-chlorophyll *a* ratio always exceeded 0.6, which is indicative of diatom-dominated microalgal communities [35]. Concentrations of chlorophyll degradation products (i.e. pheophorbides and pheophytins) were on average 6-fold lower than concentrations of intact chlorophyll *a*, which suggests the presence of an active microphytobenthic community during the polar day and limited pigment degradation in the sediment during the polar night [36]. The total pigment concentrations varied seasonally between <0.5 μg g^−1^ dry sediment during polar night and up to 3.8 μg g^−1^ during polar day. Replicate sediment samples collected during polar day showed considerable heterogeneity, pointing toward a patchy distribution of settled phytoplankton aggregates and/or benthic microalgae. In contrast, total organic carbon and total organic nitrogen contents in the bulk sediment did not fluctuate across seasons and among replicates within a season (Fig. 1B-C).

### Higher seasonal variability in bacterial diversity and community composition in the porewater and loosely attached fractions compared to the firmly attached fraction

**Figure 2:**
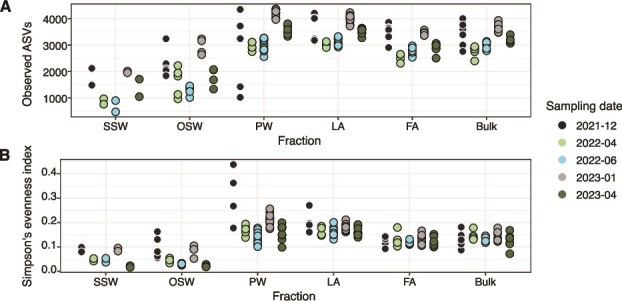
Diversity in surface (SSW) and overlying seawater (OSW), sediment fractions (PW = porewater; LA = loosely attached; FA = firmly attached), and bulk sediment (Bulk). (A) Richness based on the observed number of amplicon sequence variants (ASVs). (B) Evenness based on the Simpson’s evenness index. Sequences were subsampled to the minimum number of sequences (10 113 sequences) in the entire dataset. Each dot represents one replicate sediment grab and is colored according to the sampling date.

**Figure 3:**
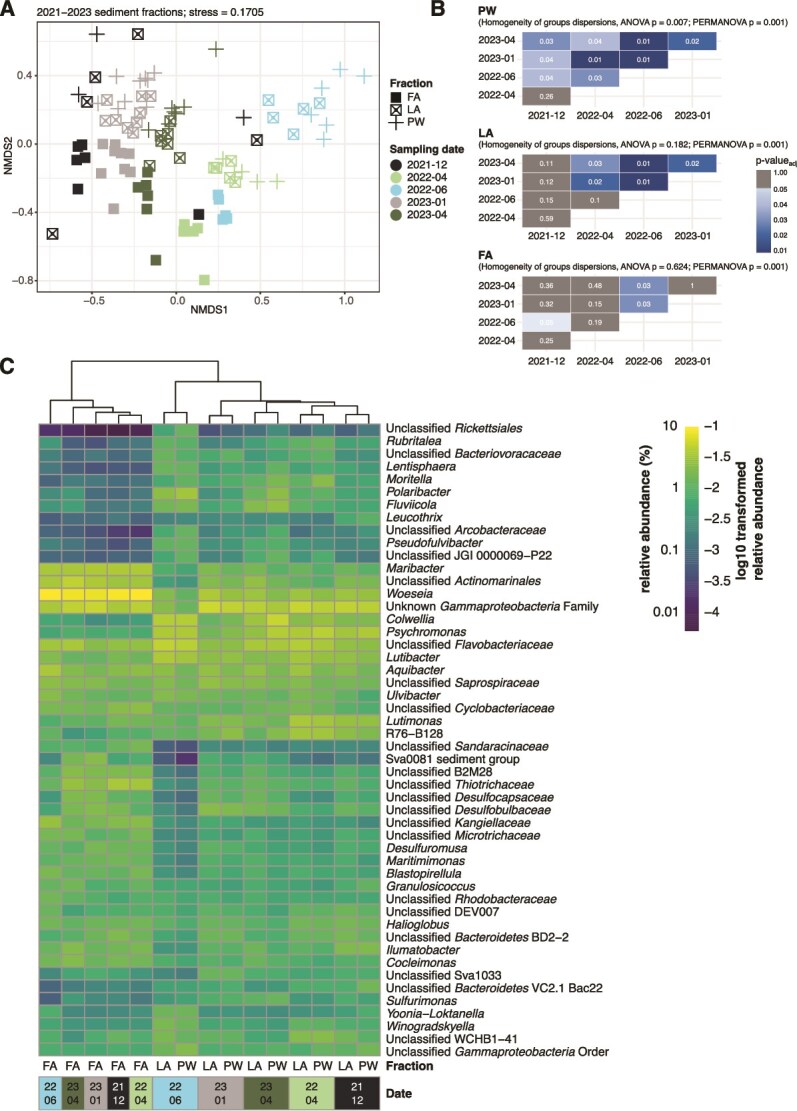
Community composition of fractions across seasons based on 16S rRNA amplicon sequencing. (A) Nonmetric multi-dimensional scaling (NMDS) plot of sediment fractions. Shapes represent the fractions; colors represent the sampling date. An NMDS plot including bulk sediment samples and with sample labels is shown in Fig. S3. (B) Pairwise PERMANOVA *P*-values (Bonferroni corrected) within fractions and across sampling dates. *P*-values from the ANOVA of betadisper results (test for homogeneity of within-group dispersion), as well as the PERMANOVA for each fraction are also reported. (C) The 20 most abundant genera per fraction and sampling date. The relative abundance of each genus for a specific date and fraction (mean of 4–8 replicate grabs collected on two sampling days) was used to select the most abundant genera.

Across seasons and in all samples, richness increased during the polar night, and decreased during the polar day (Fig. 2A). Significant seasonal changes in richness were observed for all sample types (Table S4), with the exception of surface seawater, where the limited number of replicates precluded robust statistical inference. In addition, only the OSW and PW bacterial communities showed significant seasonal shifts in community evenness and relative ASV dominance, as indicated by the Simpson’s evenness index (Fig. 2B, Table S4). Within the sediment fractions, the PW and LA had the more seasonally-dissimilar community composition (Fig. 3A, Fig. S3) compared to the FA. This observation is corroborated by Bonferroni-corrected pairwise PERMANOVA P-values (Fig. 3B), which show that the community composition in the PW and LA fractions varied more strongly across seasons compared to the FA. Notably, group dispersion differed significantly for the PW communities, indicating that some of the observed differences may in part reflect variability in dispersion between sampling dates. Nevertheless, the PERMANOVA results provide strong evidence for a significant seasonal shift in community structure. To explore potential drivers of these seasonal shifts, we performed a redundancy analysis (RDA) using total pigment content and chloroplast sequence relative abundance as proxies for organic matter input, along with sediment temperature, porewater DIC, and sampling year (Fig. S4). This analysis indicated that sediment temperature and sampling year were the variables most strongly correlated with community variation in the RDA. In contrast, pigment and chloroplast gradients explained comparatively less of the variation. The lack of a strong organic matter-specific signal may also reflect unmeasured sources of variability, such as fine-scale microenvironment heterogeneity (e.g. patchy organic matter deposition, microscale oxygen gradients, etc) that are not captured by the environmental parameters included in the RDA.

The greater seasonal variability in the PW and LA fractions was also apparent when the 20 most abundant genera in each fraction and at each sampling date were considered (Fig. 3C). During the polar day, *Rubritalea* spp., *Polaribacter* spp.*, Colwellia.* spp.*,* and *Fluviicola* spp. had particularly higher relative abundance in the PW and LA. Across sampling dates, the June 2022 (polar day summer) samples showed the most changes in the PW and LA fractions, with *Lentisphaera* spp., *Yoonia*-*Loktanella* spp., and *Winogradskyella* spp. having higher relative abundances, and a lower relative abundance of taxa involved in sulfur cycling such as Sva0081 sediment group, *Desulfocapsaceae and Desulfobulbaceae*. The genera *Woeseia* and *Maribacter,* as well as the families *Thiotrichaceae, Sandaracinaceae, and Kangiellaceae* were consistently more abundant in the FA fraction than in the PW and LA fractions across all seasons. An overview of genera with mean relative abundance ≥2% at any sampling date and in any fraction are displayed in Fig. S5. Hierarchical clustering of the samples based on the abundant genera showed a unique grouping among the FA samples regardless of season, while the PW and LA samples were intermixed based on the season (Fig. 3C). This clustering demonstrates that the dominant taxa in the FA fraction were more stable in relative abundance across seasons compared to the PW and LA. Similarly, cell numbers in the FA fraction remained relatively constant across the seasons, whereas the average cell numbers in the PW and LA fractions during the polar day were significantly higher by 1.5-fold compared to the average polar night values (*t*-test *P*-value = 0.011 for LA; *P*-value = 0.016 for PW; Table S5, Fig. S6). In terms of percentages among the sediment fractions, cells in the combined PW and LA fractions comprised 10% of the total cells during the polar night, and 15% during the polar day and conversely, the FA fraction comprised 90% during the polar night and 85% during the polar day (Fig. S7). In contrast to all sediment samples, seasonal changes were most pronounced in the SSW and OSW with a 2.8-fold increase in mean cell abundances from polar night to polar day (Fig. S6).

**Figure 4:**
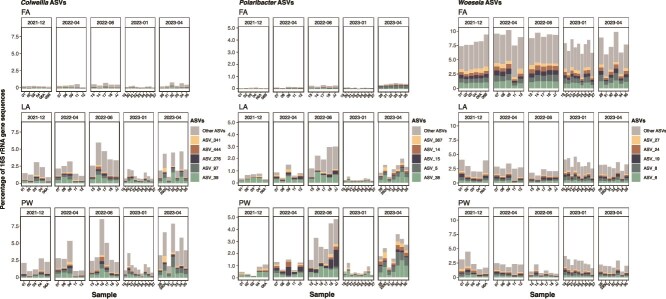
ASV relative abundances of selected genera for 16S rRNA datasets across fractions and seasons. The five most abundant ASVs across fractions and seasons are shown. Each bar represents one replicate grab sample; *x*-axis labels indicate the sediment grab number. Note the different *y*-axis scales for each taxon.

Across seasons, ASV-level responses could be observed for some abundant genera in the PW and LA fractions, but not in the FA fraction (Fig. 4). In the PW and LA fractions, most *Colwellia* ASVs had lower relative abundances during the polar night while in the polar day spring and summer, a few ASVs had at least 2-fold higher relative abundance. In the FA fraction, however, the sum of relative abundances of all *Colwellia* ASVs were consistently below 1% across the seasons. The genus *Polaribacter* also had higher relative abundance in the polar day summer in the PW and LA fractions, and ASVs within this genus fluctuated in relative abundance across seasons. Lastly, unlike the other selected genera, *Woeseia* ASVs did not fluctuate in relative abundance in all seasons and fractions. From the abovementioned genera, a few ASVs that increased in the fractions also increased in the overlying seawater (Fig. S8). For instance, *Polaribacter* ASV 15 was the most abundant in the seawater samples in the summer. This ASV also had higher relative abundance in the PW fraction at the same sampling date. Relative abundances of all ASVs across seasons are reported in Table S6.

### Higher abundance of *Gammaproteobacteria* as polar day progresses, while *Deltaproteobacteria* showed higher abundance during polar night

**Figure 5:**
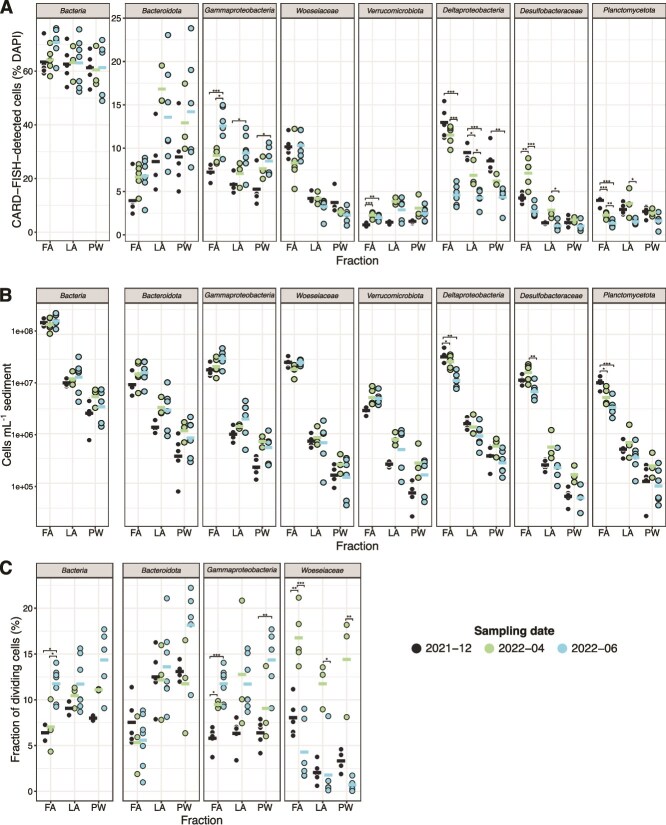
*In situ* cell abundances and fraction of dividing cells of major taxa. (A) Abundance of major taxa shown relative to total cell counts, determined by CARD-FISH. (B) Absolute abundance of major taxa in cells ml^−1^ sediment. Percentages and absolute abundances obtained from CARD-FISH are detailed in Table S7. (C) Fraction of dividing cells for selected taxonomic groups. Data from April 2022 have been described previously in [25]. Each dot represents one sediment grab. The solid horizontal bars represent the mean. Within one fraction, statistically significant differences between seasons were determined by one-way ANOVA followed by pairwise *t*-tests with Bonferroni correction for normally distributed data and Kruskal–Wallis followed by Dunn’s test with Bonferroni correction for non-normally distributed data. *P*-value: 0–.001 “^***^”, .001–.01 “^**^”, .01–.05 “^*^.” January and April 2023 fractions were not processed for CARD-FISH and fraction of dividing cells measurements.

In all the fractions, *Bacteroidota, Gammaproteobacteria*, and *Verrucomicrobiota* showed a nearly 2-fold increase in relative abundances during the polar day compared to the polar night (Fig. 5A, Table S7). Absolute numbers of these taxa also increased (Fig. 5B), but the increase was not statistically significant, likely due to sample heterogeneity between sediment grabs. In contrast, *Planctomycetota* showed an opposite trend, with significantly higher absolute and relative abundance in the FA during the polar night. Similarly, *Deltaproteobacteria* showed a statistically significantly higher relative abundance during the polar night in all the fractions (mean relative abundance in FA: 13%; LA: 10%; PW: 9%) compared to the polar day summer (5% in all the fractions). The absolute cell numbers of *Deltaproteobacteria* were also significantly higher in the FA during the polar night than in the polar day spring and summer. The deltaproteobacterial *in situ* cell numbers are in line with the relative abundance based on 16S rRNA gene amplicon data, with most of the changes belonging to orders *Desulfobulbales* and *Desulfuromonadales* (Fig. S9). Within the *Deltaproteobacteria*, the family *Desulfobacteraceae,* however, had the highest absolute and relative abundance in polar day spring for both FA and LA. Finally, the *Woeseiaceae*, a prominent group in the FA (8%–10% of total cells), showed unchanged absolute and relative cell abundances across all fractions and seasons.

There were notable differences when comparing the CARD-FISH relative abundances to the 16S rRNA relative abundances, largely explained by variations in 16S rRNA gene copy numbers across taxa. For instance, 16S sequencing showed higher relative abundances for *Planctomycetes*, *Gammaproteobacteria*, *Bacteroidota*, and *Verrucomicrobiota*, aligning with known 16S rRNA gene copy numbers for these taxa [37]. In contrast, the ratio of relative 16S rRNA gene read abundances determined by 16S rRNA sequencing and relative cell numbers determined by FISH for *Woeseiaceae* was 0.8 ± 0.2. This near 1:1 relationship is consistent with the presence of a single 16S rRNA gene copy in *Woeseia oceani* (*rrn*DB; [38]) and related *Woeseiaceae* MAGs (Tomeu Viver, personal communication).

In terms of the fraction of dividing cells, members of the domain *Bacteria* in the PW, LA, and FA generally had the highest proportion of dividing cells during the polar day summer (Fig. 5C; 6%–9% of cells in December 2021 vs. 12%–14% in June 2022). Likewise, *Gammaproteobacteria* showed higher fractions of dividing cells in all sediment fractions during summer, with statistically significant increases in the PW and FA. This pattern is consistent with the 1.5- to 2-fold higher absolute abundances of this taxon observed in this season compared to the polar night. Notably, *Bacteroidota* in the PW showed the highest fraction of dividing cells across all fractions, with an average of 18% of *Bacteroidota* cells dividing during the polar day summer. During the polar night and the polar day spring, the fraction of dividing cells for this taxon in the PW samples was between 12% and 13%. The most pronounced changes in the fraction of dividing cells between the polar day and the polar night were observed for the *Woeseiaceae*. During the polar day summer, this taxon exhibited a substantially lower fraction of dividing cells—4-fold lower in the FA, 6-fold in the LA, and 14-fold in the PW—compared to the polar day spring. Despite the high fraction of dividing cells in spring, cell abundances did not increase, potentially indicating top–down mortality, e.g. by viruses as discussed previously [39]. During the polar night, the fraction of dividing *Woeseiaceae* cells remained higher in the FA fraction (8%) compared to the PW and LA (2%–3%).

### Higher laminarin extracellular hydrolysis rates during polar day in the porewater and loosely attached cell fractions

**Figure 6:**
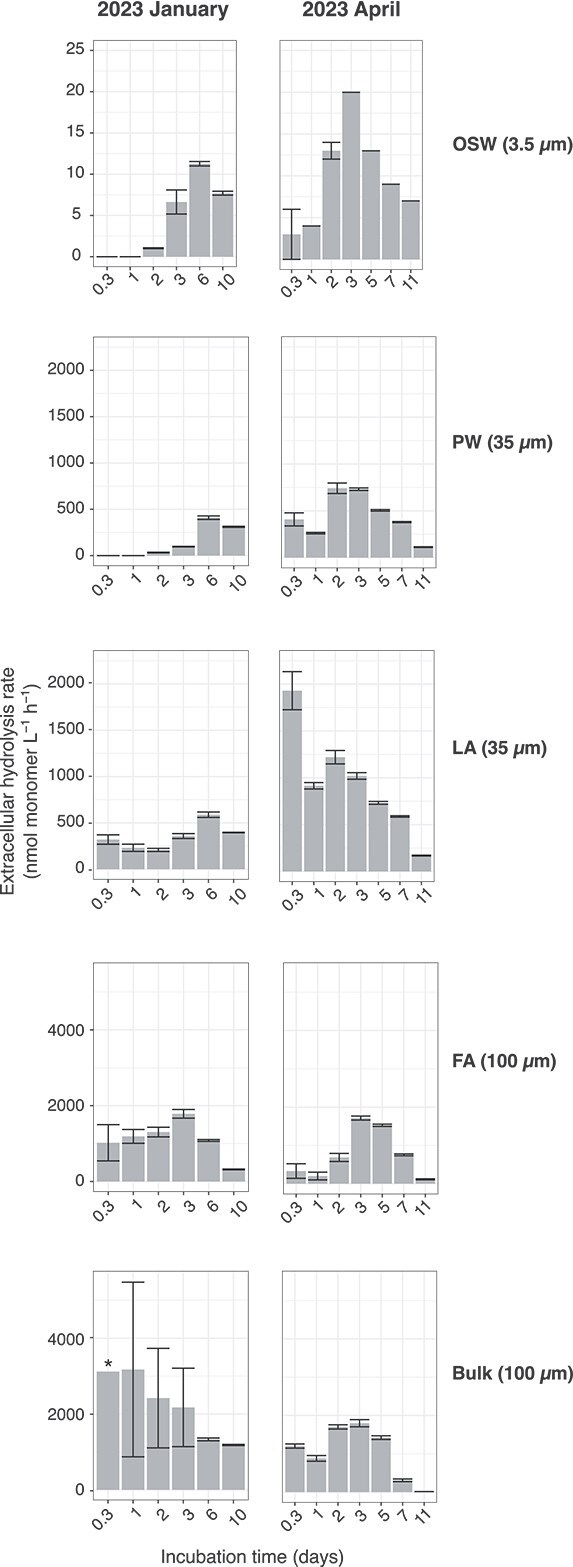
Mean extracellular hydrolysis rates of FLA-laminarin in the overlying seawater, sediment fractions, and bulk sediment from the January and April 2023 incubations. The final concentrations of added FLA-laminarin are indicated in parentheses. The error bars show the standard deviation of triplicates. The asterisk indicates a very high standard deviation for the triplicates (bulk 100 μM from January 2023; mean: 3118.5 nmol monomer l^−1^ h^−1^; SD: 3133.4 nmol monomer l^−1^ h^−1^). Note the different *y*-axis scales. April 2023 data have been described previously in [25]. Note that the measured rates are considered potential rates because the presence of laminarin in the environmental samples could compete with the added fluorescently labeled polysaccharides for enzyme active sites. Rate data for December 2021, April 2022, and June 2022 are shown in Fig. S10.

The sampling site in Isfjorden is characterized by primary production dominated by diatoms, as indicated by the sediment photopigment data (Fig. 1A). The major storage polysaccharide in diatoms is laminarin [40]; hence, we measured the extracellular hydrolysis of this polysaccharide in the fractions, bulk sediment, and overlying seawater across the seasons. In incubations of PW collected from the polar night (January 2023), hydrolysis was first measurable after 2–3 days, and maximum rates were reached after 6 days of incubation (Fig. 6). In contrast, during the polar day spring (April 2023), laminarin hydrolysis was measured after only 8 h, with highest rates observed after 2–3 days. Furthermore, the maximum laminarin hydrolysis rates in the PW incubations were significantly higher (*P*-value = .0002) by almost 2-fold in samples collected during the polar day spring compared to those from the polar night. In the LA incubations, the hydrolysis rates peaked within 8 h in the polar day spring samples and were significantly higher, by a factor of three, than those measured in incubations of the polar night samples (*P*-value = .0003). In the FA incubations, maximum rates measured in incubations of polar night samples were as high as those in samples collected during the polar day spring (*P*-value = .38). For FA samples from both seasons, hydrolysis was observed after 8 h and the maximum rate was detected after 3 days.

In the OSW samples collected during the polar night, hydrolysis was first measurable after 2 days of incubation with maximum rates observed after 6 days whereas in samples from polar day spring, laminarin hydrolysis could already be measured after 8 h of incubation with 2-fold higher maximum rates (*P*-value = .00001) already detected after 3 days. Laminarin extracellular hydrolysis measurements were also done with samples from December 2021, April 2022, and June 2022 and resulted in similar seasonal hydrolysis patterns (Fig. S10).

### Per-cell oxygen consumption rates remained stable throughout the seasons

**Figure 7:**
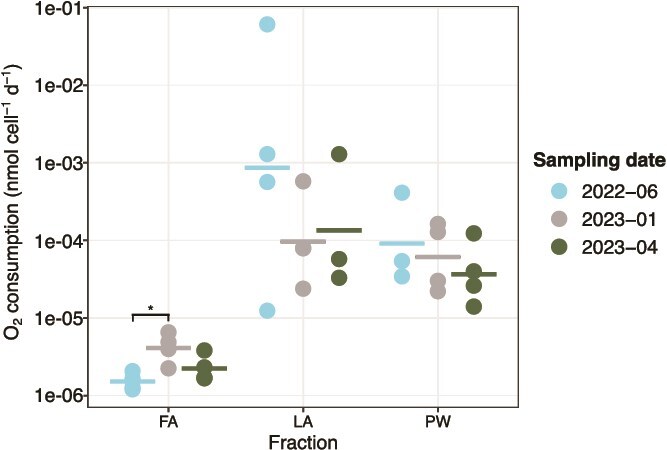
Per-cell oxygen consumption in sediment fractions across sampling dates. The horizontal bars represent the mean of the replicates. Data from April 2023 have been described previously in [25]. For June 2022, each dot represents measurements from one sediment grab. For January and April 2023, each dot represents the mean of 1–3 technical replicates from one sediment grab. Asterisk indicates *P*-value <.05. Within one fraction, statistical significance was determined by one-way ANOVA followed by pairwise *t*-tests between seasons with Bonferroni correction. Oxygen consumption in the fractions was not measured in December 2021 and April 2022.

Within the PW and LA fractions, the cell-normalized oxygen consumption rates did not differ significantly across seasons, but the per-cell rates in the PW and LA fractions were consistently 1–2 orders of magnitude higher than in the FA fraction (Fig. 7). The FA fraction showed the lowest per-cell oxygen consumption rates across seasons (1.2 × 10^−6^ to 6.6 × 10^−6^ nmol cell^−1^ d^−1^). The per-cell O_2_ consumption in June 2022 was significantly lower than in January 2023. As CO_2_ is produced during respiration, the observed seasonal stability of O_2_ consumption in the PW is consistent with the seasonally unchanged PW dissolved inorganic carbon (DIC) concentrations (Fig. S11), although PW DIC may also be strongly controlled by seawater flushing.

## Discussion

In this study, we build upon our previous work demonstrating that bacterial fractions—defined as cells in the porewater, loosely attached, or firmly attached to sediment grains—differ in both composition and activity in Isfjorden sandy surface sediments [25]. Here, we leveraged the extreme seasonality in the Arctic as a natural experiment wherein the benthic community receives organic matter from primary production during polar day, and conversely, no new input is received from photosynthesis during polar night (even if winter activity is still likely sustained by organic matter produced during the previous productive season). The seasonal shifts in primary production were reflected by high pigment concentrations in April, consistent with spring bloom dynamics in Svalbard fjords, where primary production typically peaks between April and May [41–43]. Our community-based observations and rate measurements demonstrate that the bacterial fractions respond differently to the extreme seasonality in Isfjorden. Specifically, we observed a more pronounced seasonal change in the porewater and loosely attached bacterial communities than in the firmly attached community, as indicated by both significant shifts in community composition and laminarin hydrolysis rates. Although the firmly attached cell fraction also showed seasonal variations, its community was more stable than those in the other fractions. This contrast is likely driven by the microenvironment of the bacteria, specifically their location on the grain, which influences the amount of substrates they encounter and the cells’ susceptibility to physical disturbances or mechanical abrasion. Here, we discuss the contrasts between the fractions and how they respond to the extreme seasonal changes in Isfjorden. The porewater and loosely attached fractions will be referred to collectively as PW-LA and discussed together, since both fractions exhibited similar compositional and activity patterns.

### Seasonally responsive porewater and loosely attached fractions

**Figure 8:**
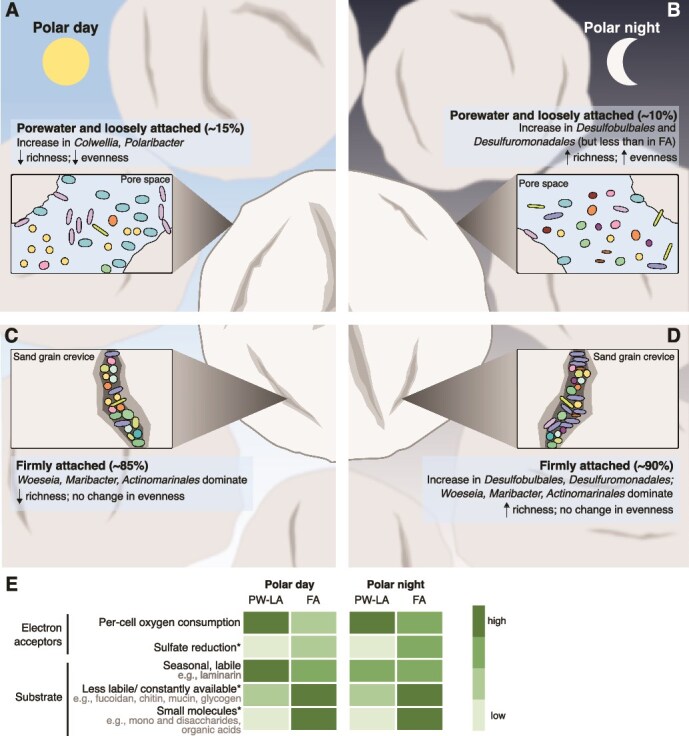
Seasonal responses of bacterial communities in sediment fractions. (A) Community changes in the PW-LA during polar day and (B) polar night. (C) Community changes in the FA during polar day and (D) polar night. (E) The preferred electron acceptors and potential substrates between fractions and seasonal extremes. Asterisk indicates processes inferred from the presence of taxa. Supporting data and literature on the potential substrates are detailed in Table S8.

The PW-LA communities during the polar day showed significantly higher laminarin hydrolysis rates and a larger dominance of heterotrophic bacterial taxa, such as *Colwellia* and *Polaribacter,* that are known to readily respond to an increase in labile organic matter [44, 45] (Fig. 8A). The growth of a few heterotrophic genera was mirrored in the decrease in evenness in both fractions, which was statistically significant in the PW. These findings point to the selection of boom-and-bust specialists that can rapidly take advantage of fresh organic matter supply during the polar day, similar to the selection of copiotrophs and/or fast-growing heterotrophic bacteria in the water column during spring blooms [46, 47]. The significant changes in the community composition and the extracellular laminarin hydrolysis rates in the PW-LA compared to the FA fraction indicate that the seasonal variability in labile organic matter supply has a stronger effect on cells in the porewater and on those loosely attached to sediment grains. This contrast likely reflects the influence of enhanced advective flow in the PW-LA, increasing these fractions’ access to oxygen and substrates, as opposed to the more diffusion-limited environment occupied by the FA fraction, as previously proposed [25]. To visualize ripple migration (indicative of advective transport in the sediment), we deployed a camera on the seafloor at the station in December 2021 (retrieved after 3 days) and again in April 2022 (retrieved in June 2022 after 2 months). In both deployments, we observed ripples on the sediment, which were frequently reworked and resuspended via currents or storms over the course of a few days (Fig. S12 and Supplementary video in [25]). Porewater advection is generally driven by horizontal pressure gradients at the sediment surface, which arise when bottom water flow is deflected by topographic features such as ripples [48]. Our macro-scale observations support the predominance of advection at the site, enhancing solute exchange between sediments and overlying water, thereby facilitating O_2_ supply particularly on exposed grain surfaces. These observations also indicate that the layers below the measured oxygen penetration depth were likely not permanently anoxic, due to constant sediment reworking.

During the polar night, the PW-LA fractions constituted a significantly lower proportion of the total cells in the bulk community, showed unchanged per cell oxygen consumption rates, but had significantly lower extracellular laminarin hydrolysis rates compared to the polar day (Fig. 8B). The clear seasonal difference in extracellular laminarin hydrolysis rates in the PW-LA fractions is in agreement with previous observations of reduced gene expression for laminarin-hydrolyzing enzymes during the polar night [18]. Bacterial communities in the PW-LA fractions likely sustain respiration during the polar night by utilizing alternative substrates or electron donors, such as recalcitrant organic matter or animal-derived organic matter (e.g. mucin), rather than fresh, labile organic matter. The use of different substrates during the polar night goes along with a decrease in the positive selection of rapidly-growing taxa specialized in degrading labile organic matter and therefore results in a more even community (Fig. 2B). Overall, these patterns suggest that during the polar night, the PW-LA communities potentially utilize a broader spectrum of organic substrates, leading to reduced enzymatic activity toward highly labile compounds such as laminarin and a shift toward a more even community until primary productivity resumes with the return of the polar day.

### Buffered response in the firmly attached fraction

Across seasons, both the community composition and the extracellular laminarin hydrolysis rates in the FA fraction remained comparatively stable, showing no significant changes relative to the PW-LA fractions. Since the FA fraction comprised 85%–90% of the total cells in Isfjorden sandy surface sediments, this result likely explains the lack of seasonal fluctuations in earlier investigations of bulk sediment bacterial communities at the same station in Isfjorden [2, 18]. In all seasons, *Woeseia* was the most abundant genus in the FA fraction, and seasonal changes at the ASV level within the genus could not be detected. Moreover, *Maribacter* spp. and an unclassified member of *Actinomarinales* also remained consistently abundant in this fraction (Fig. 8C and D). These taxa have been associated with laminarin degradation [49–52] and show capacities for degrading other polysaccharides [49–53]. The seasonal stability of these potential laminarin-degrading taxa, along with other laminarin degraders, as well as the stable total cell numbers, may explain the lack of statistically significant differences in the laminarin hydrolysis rates in the FA fraction across seasons. Moreover, the consistently high relative abundance of *Kangiellaceae* and *Sandaracinaceae* in the FA fraction suggests that the uptake of hydrolysis and fermentation products (potentially derived from constantly available substrates, e.g. glycogen from necromass, peptidoglycan from bacterial cell walls, or mucin from bioturbating fauna) may be occurring in this fraction. Members of the family *Kangiellaceae* have been shown to predominantly rely on amino acids as carbon and nitrogen sources [54, 55]. *Sandaracinaceae* belongs to the phylum *Myxococcota,* which shows genomic potential for consuming low molecular weight organic matter and fermentation products [56], as well as the potential for exhibiting predatory behavior [57]. The largely unchanged relative abundance of both low- and high-molecular-weight organic matter degrading taxa in the FA fraction suggests its potential to utilize diverse substrates year-round, contributing to its overall seasonal stability. Moreover, the high diversity and cell abundance in this fraction point toward a vast array of enzymes that can work in concert to degrade diverse substrates [58].

During the polar night in the FA fraction, we observed significantly higher oxygen consumption rates, an increase in community richness but no change in evenness, and a significantly higher cell abundance of *Deltaproteobacteria* compared to the polar day (Fig. 8D). The higher abundance of *Deltaproteobacteria* (mostly *Desulfobulbales and Desulfuromonadales*, both strictly anaerobic taxa [59, 60]), mainly involved in sulfur or iron cycling [59, 61, 62], indicate the prevalence of anoxic microenvironments in the absence of benthic photosynthesis and an increased importance of sulfur and iron cycling during the polar night. Sulfate reduction has been shown to be the dominant anaerobic carbon remineralization pathway in Svalbard fjord sediments, accounting for at least 50% of the total carbon remineralization [17, 63]. Although the higher oxygen consumption rates may seem contradictory to the increased importance of sulfate reduction during the polar night, the higher rate of oxygen consumption could deplete oxygen in locations where diffusive forces would dominate transport, leading to anoxic microniches where sulfate reduction can occur [64, 65]. In any case, the continuous carbon cycling activities in the FA fraction demonstrate that the bacterial community remains active, potentially sustaining itself through the recycling of less labile organic matter or the use of constantly available substrates such as microbial necromass [66–68]. In line with this idea, a glycoside hydrolase family 23, annotated as a peptidoglycan lyase, was highly expressed year-round and was upregulated in winter at the same station in Svalbard, indicating that recycling of bacterial cell walls could be an important process sustaining the benthic community [18]. Moreover, in Isfjorden surface sediments, the estimated residence time of organic carbon ranged between 1 and 2 years, based on our measurements of bulk sediment oxygen consumption rates and total organic carbon content. Previous investigations of sediment cores from Young Sound (Greenland) incubated at *in situ* temperatures without carbon addition also demonstrated stable carbon oxidation rates for 400 days [69]. These results suggest that carbon oxidation is sustained by a substantial pool of relatively inert organic matter with a half-life of over 1 year. Stable baseline remineralization processes independent of fresh organic matter input are likely mediated by the firmly attached fraction of bacteria in sediments, as evidenced by this fraction’s community composition and seasonally stable cell numbers. Nevertheless, in the FA, the fraction of dividing *Gammaproteobacteria* and *Woeseia* cells changed significantly between winter and spring, despite stable ASV relative abundances and absolute cell numbers. Investigating changes in single-cell activity or gene expression within this fraction could further elucidate finer-scale seasonal responses among cells in this fraction.

### Benthic microbiome partitioning in organic matter degradation

From our findings, we propose the following main paths for organic matter degradation in Isfjorden surface sediments (Fig. 8E, Table S8): During polar day, advective flow delivers oxygen and organic matter derived from primary production into the pore space. This process selects for aerobic taxa that are specialized in degrading labile organic matter in the PW-LA fraction. During the polar night, heterotrophic activity likely shifts toward the hydrolysis of less labile or constantly available substrates, yielding low-molecular-weight products that can be shared with other members of the microbial community that lack extracellular enzymes [70]. Metagenomic data suggest that all fractions have the potential to degrade less labile organic matter like fucoidan and consistently available substrates, such as mucin, chitin, glycogen, and peptidoglycan [18, 25]. Furthermore, in winter, the increase in sulfur cycling taxa, particularly in the FA fraction, potentially indicates the utilization of low-molecular-weight organic matter by sulfate reducers in this fraction. The patterns observed here further highlight that the classical cascade of electron donors and acceptors is not limited to sediment depth zonation [71]. It also occurs within microniches in the top 2 cm of sandy sediments, where multiple electron acceptors such as oxygen, sulfate, and nitrate are present. This finer-scale redox zonation has previously been demonstrated using radiotracer experiments on silty and clayey sediments [64], as well as in chemostat incubations of sandy sediments [72].

## Conclusion

By employing multiple complementary methods and fractionating benthic communities, we gained new insights into how microbial communities respond to the pronounced seasonality in the Arctic. Our data show a partitioning of the benthic bacterial community into seasonal and stable heterotrophic guilds: the porewater and loosely attached cells, although a minor fraction in terms of cell numbers, constituted the seasonally responsive communities, which may play an important role in bentho-pelagic coupling. In contrast, the FA fraction, comprising the majority of cells, exhibited greater seasonal stability, indicating it likely plays a central role in maintaining baseline recycling processes year-round, regardless of seasonal fluctuations in substrate availability. Its diverse metabolic capabilities potentially enable this fraction to buffer the system by remineralizing not only seasonal and labile organic matter but also less labile or constantly available substrates. The potential differentiation in substrate specialization between the PW-LA and FA fractions observed in Arctic surface sediments may also apply to other coastal sandy sediments where advection is a dominant process. In temperate regions, for example, organic matter availability continues to vary seasonally, though typically less extremely than in the Arctic [73–75]. In these coastal regions, the PW and LA fractions could similarly represent the more responsive and dynamic communities, whereas the FA fraction may maintain a comparatively stable composition and activity. Collectively, these findings provide a novel mechanistic understanding of the resilience of benthic microbial communities, maintaining functional stability despite varying organic matter input.

## Supplementary Material

Moncada_et_al-SupplementaryInformation_ycaf161

Moncada_et_al-SupplementaryInformation_Tables_ycaf161

## Data Availability

Sequences have been deposited to the European Nucleotide Archive (ENA) under accession numbers PRJEB67636 (April 2022 data), and PRJEB86178 (December 2021, June 2022, January 2023, and April 2023 data).
