# Supplementary material for: Seasonal and stable heterotrophic guilds drive Arctic benthic microbiome functioning across polar day and night
Source: ISME Commun. 2025 Sep 19;5(1):ycaf161. doi: 10.1093/ismeco/ycaf161 (PMC12503162; doi:10.1093/ismeco/ycaf161)
Supplement: Moncada_et_al-SupplementaryInformation_ycaf161 [file moncada_et_al-supplementaryinformation_ycaf161.pdf]

Supplementary information

Seasonal and stable heterotrophic guilds drive Arctic benthic microbiome functioning across polar day and night

Chyrene Moncada, Carol Arnosti, Jan D. Brüwer, Dirk de Beer, Gunter Wegener, Peter Stief, Marit R. van Erk, Jürgen Titschack, Rudolf Amann, Katrin Knittel

SUPPLEMENTARY MATERIALS AND METHODS

Sediment grain size measurements

Particle size measurements were done using a Beckman Coulter Laser Diffraction Particle Size Analyzer LS 13320 (Beckman Coulter, Krefeld, Germany) as described previously [1] and following the principles of the methods established in Boehnert et al [2]. Prior to analysis, bulk sediment was boiled with approximately 0.3 g tetra-sodium diphosphate decahydrate ($Na_4P_2O_7 \times 10H_2O$) for 3 minutes to separate the aggregates.

Pigment analysis

Analysis of bulk sediment pigment content was done according to the method described in Meier et al [3]. Briefly, approximately 10 mL sediment was freeze-dried in an Alpha 1-2 LDplus freeze dryer (Martin Christ Gefriertrocknungsanlagen GmbH, Osterode am Harz, Germany) and 0.5 g sediment (dry weight) was used for further extraction. Extraction was performed via two rounds in 1 ml ethanol/acetone/water (45:45:10) solution in a 4°C ultrasonic bath for 90 min per run. The solution was centrifuged for 4 min at 16,000 × *g* (Eppendorf 5415C centrifuge, Wesseling-Berzdorf, Germany) and the supernatant was collected and filtered through 0.2-µm PTFE membrane filters (WICOM Germany GmbH,Heppenheim, Germany), and stored at −20°C until analysis. Pigments were separated and analyzed on a Waters ultraperformance liquid chromatography (UPLC) H-class system (Waters, Milford, MA, USA) with an Acquity UPLC BEH C_{18} column (1.7 µm, 2.1 × 150 mm) (Waters Corporation, Milford, USA). Column temperature, solvent gradients, and flow rate were as described previously [3]. Analysis of the data was done using the Empower 3 software (Waters, Milford, MA, USA). For total concentration, standards with known concentrations were measured and response factors for all analyzed compounds were calculated.

Carbon and nitrogen content analysis

Carbon and nitrogen content from bulk sediments were measured as described previously [1]. Briefly, freeze-dried sediments were ground using a Planetary Micro Mill (Pulverisette 7, Fritsch, Idar-Oberstein, Germany). For total carbon and nitrogen analyses, ~25–50 mg of powdered sediment was packed into 5×9 mm tin capsules (HEKAtech, Wegberg Germany). Prior to organic carbon analysis, inorganic carbon was removed using 1M HCl. Samples were analyzed on a Euro EA-CNS elemental analyzer with thermal conductivity detection (HEKAtech, Wegberg, Germany). Calibration was done with 0.2–2 mg sulfanilamide standards.

To measure dissolved inorganic carbon in the seawater and porewater, the samples were sterile-filtered and headspace-free stored in 2 mL Zinsser vials with butyl septa. DIC concentrations were measured by flow injection coupled to conductivity detection [4]. Values were quantified against standards of known concentrations (0-10 mM).

DNA extraction and 16S rRNA gene amplification

To extract DNA from the December 2021 seawater, PW, and LA samples, we used the DNeasy PowerWater Kit (QIAGEN GmbH, Hilden Germany). The ZymoBIOMICS DNA/RNA Miniprep kit (Zymo Research, California, USA) was used to extract DNA from the FA and bulk sediment samples from December 2021, as well as all other samples and fractions collected until 2023. To amplify the V3-V4 region of the 16S rRNA gene, primers S-D-Bact-0341-b-S-17 and S-D-Bact-0785-a-A-21 were used [5].

Amplicon sequence variant analyses

Amplicon sequences were processed by removing barcodes and primers using cutadapt v1.15 [6], and quality trimmed with the DADA2 v1.16.0 [7] filterAndTrim function. Reads shorter than 100 bp were discarded. Amplicon sequence variants (ASVs) were inferred using DADA2 with the pool parameter set to TRUE. Taxonomy was assigned using the SILVA NR99 v138.1 database [8]. Absolute singletons were removed before diversity analyses. Community

analyses were conducted in R v4.2.1 [9]. Alpha diversity was calculated via repeated subsampling to the minimum number of sequences of one specific sample in the entire dataset (10,113 sequences) using the subsamplingNGS.R function (https://github.com/chassenr/NGS/blob/master/Plotting/SubsampleNGS.R). To quantify seasonal changes in community evenness and the relative ASV dominance, the Simpson's evenness index was used. Bray–Curtis dissimilarities and NMDS plots were generated using the phyloseq v1.42.0 R package [10] to show broader community-level patterns across seasons and fractions. To assess significant differences between fractions across seasons, we first tested homogeneity of within-group dispersion using the betadisper function, followed by PERMANOVA with the adonis2 function. When the global PERMANOVA indicated a significant effect (p-value <0.05), we conducted pairwise PERMANOVA tests to identify which seasons differed significantly within a fraction. Both betadisper and adonis2 are implemented in the vegan v2.6.4 R package [11]. Redundancy analysis (RDA) was also performed for each sediment fraction to assess relationships between Hellinger-transformed bacterial community composition and environmental variables. To avoid multicollinearity, variables with Pearson correlation > 0.8 were reduced by retaining one representative from each correlated group (e.g. total pigments for chlorophyll-a, fucoxanthin, and daylight; sediment temperature for seawater temperature and salinity; porewater DIC for both porewater and seawater DIC). RDA models were fitted using the rda function from vegan [11].

Sample processing for total cell counts

Fixed bulk sediments and FA fractions were washed three times with a 1:1 mixture of 1X phosphate-buffered saline (PBS: 13.7 mM NaCl, 0.27 mM KCl, 1 mM Na_2HPO_4, 0.2 mM KH_2PO_4, pH 7.2) and ethanol to remove excess formaldehyde, allowing particles to settle for approximately 30 s before discarding the supernatant. Cells were detached from sediment grains by sonication as previously described [1], using six rounds at 86% amplitude (0.2 s pulses for 30 s) with a Sonopuls Mini20 (Bandelin, BANDELIN Electronic GmbH & Co KG, Berlin, Germany). The sonicated cells from the FA fraction and bulk, as well as the seawater, PW, and LA samples were then filtered onto 0.2 µm polycarbonate filters (Merck, Darmstadt, Germany) and stained with 4',6-diamidino-2-phenylindole (DAPI, 1 µg mL^{-1} final concentration) in a Citifluor/VectaShield (3:1) mixture (Citifluor: CitiFluor Ltd., London, UK; Vectashield: Vector Laboratories, CA, USA)

SUPPLEMENTARY FIGURES

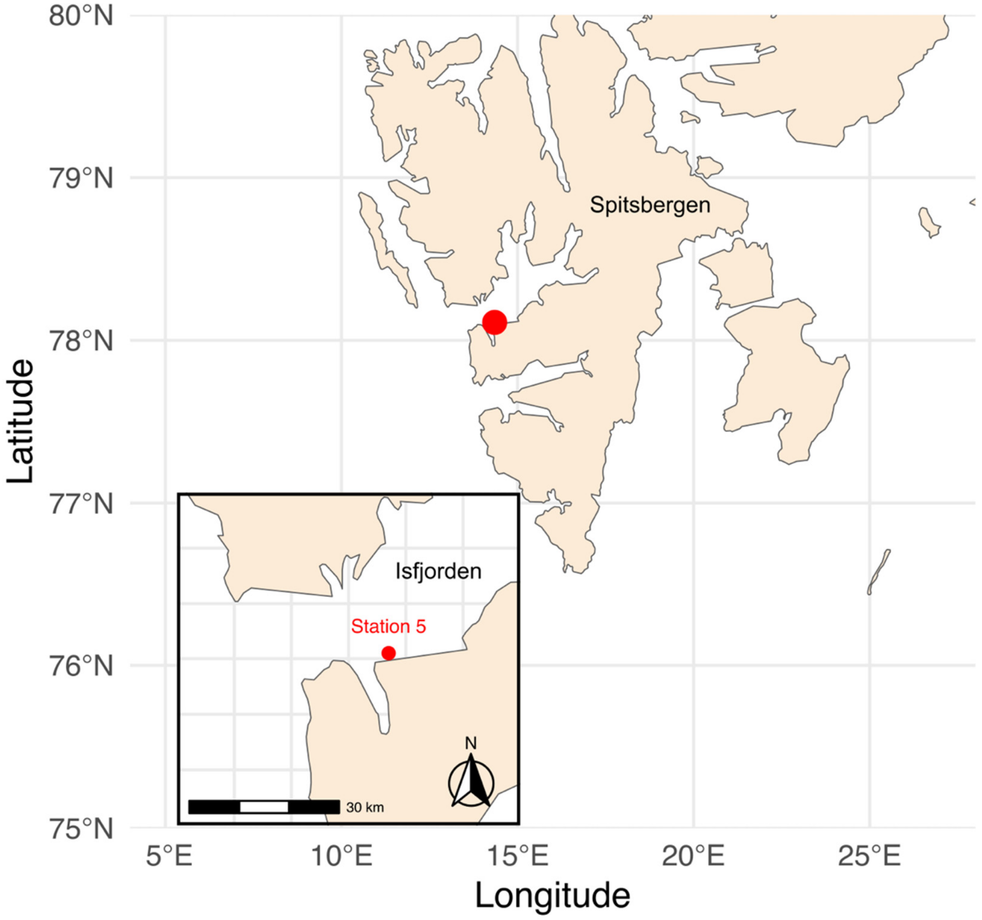

Figure S1. Map of sampling station. Samples were collected in Isfjorden, an Arctic fjord in western Spitsbergen, Svalbard.

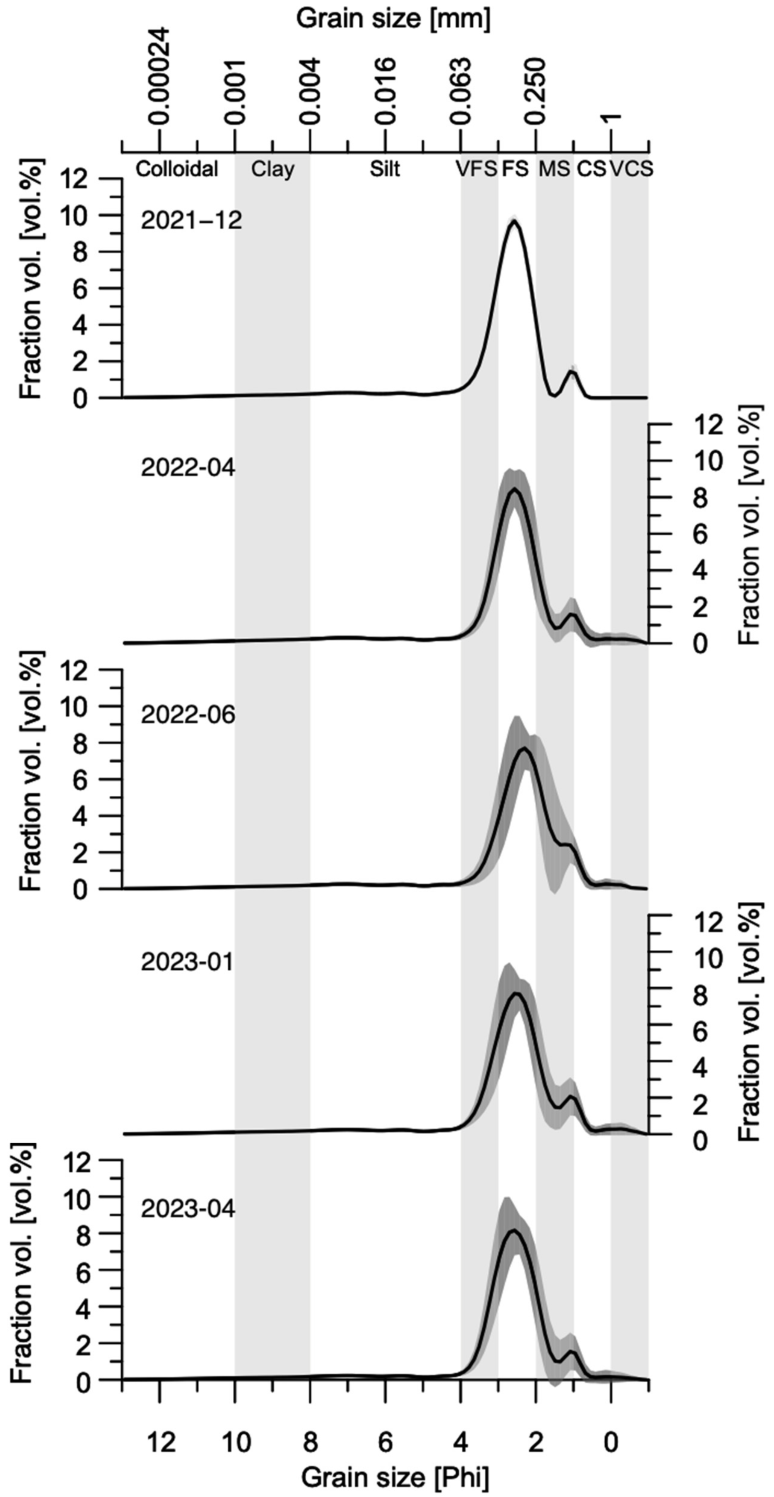

Figure S2. Grain size distribution of bulk surface sediments (top 0-2 cm) across sampling dates. VFS: very fine sand; FS: fine sand; MS: medium sand; CS: coarse sand; VCS: very coarse sand.

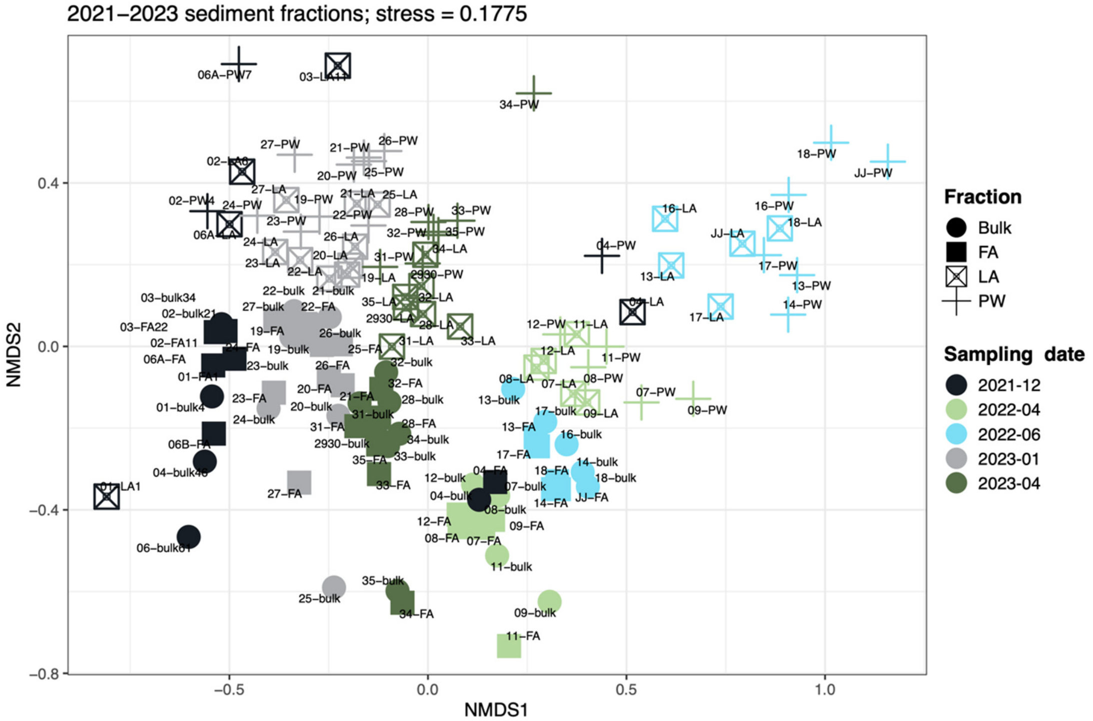

Figure S3. Non-metric multi-dimensional scaling (NMDS) plot of sediment fractions and bulk sediment with sample labels. Shapes represent the fractions; colors represent the sampling date.

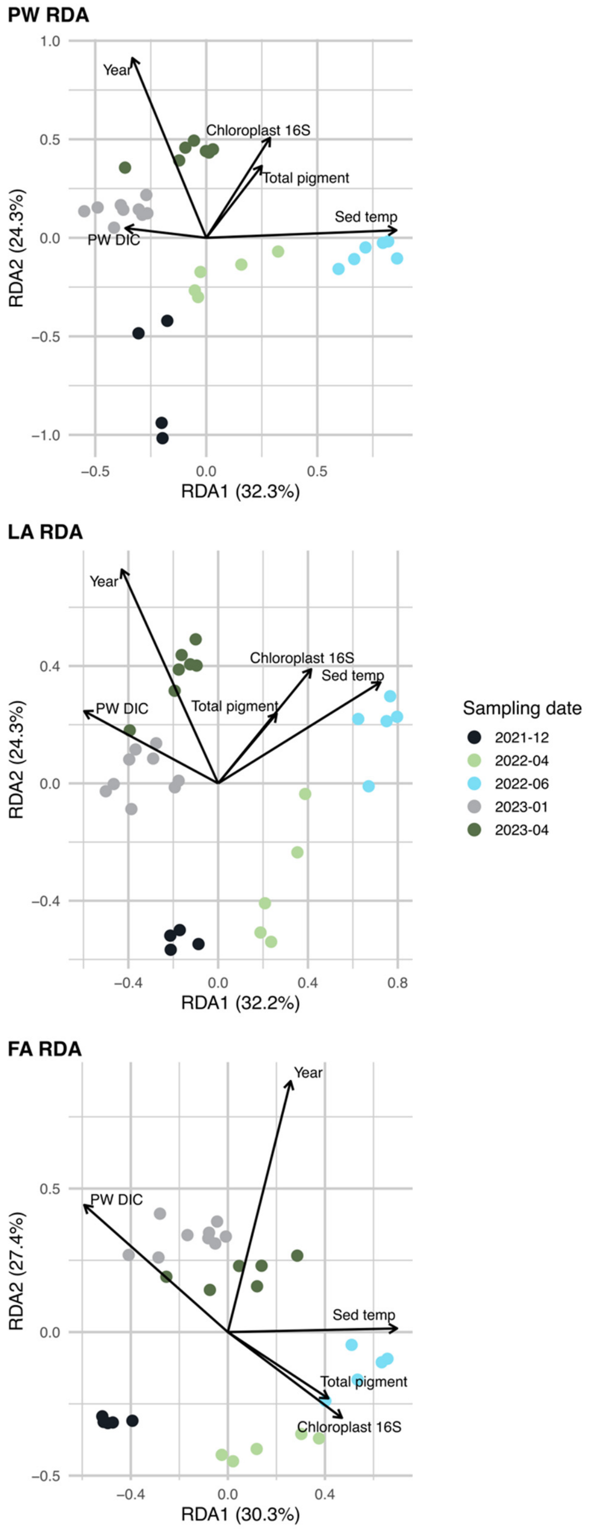

Figure S4. Redundancy analysis (RDA) biplots for the porewater, loosely attached, and firmly attached communities constrained by environmental variables. Percentages on the axes indicate the proportion of constrained variance explained by RDA1 and RDA2. Samples are colored according to sampling date. Highly collinear variables were excluded to avoid multicollinearity (see Methods).

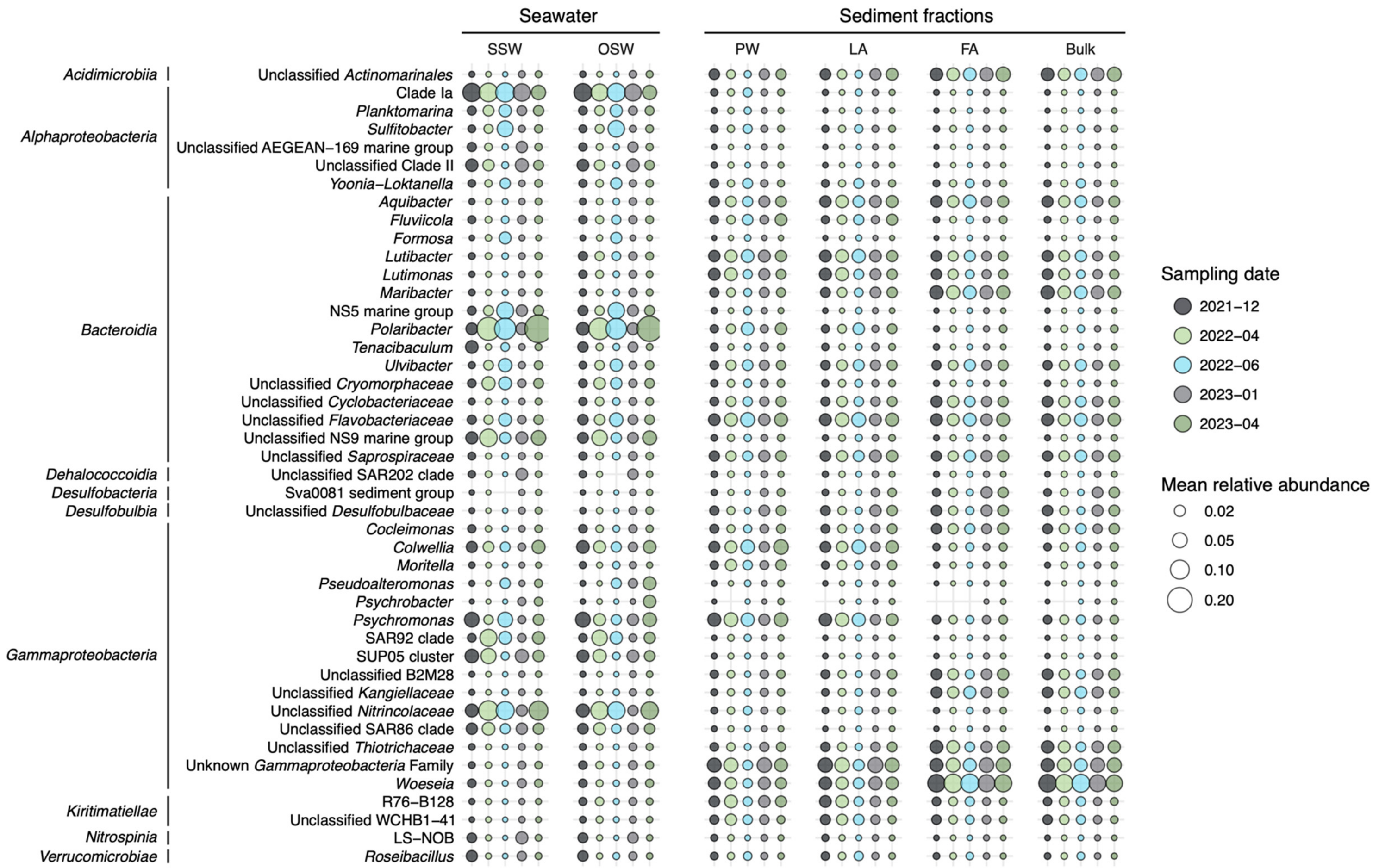

Figure S5. Community composition in Svalbard seawater and sediment fractions based on 16S rRNA gene sequencing. Only genera with a mean relative abundance of >2% of total reads in at least one sampling date and fraction are displayed. For unclassified genera, the next higher taxonomic classification is indicated. SSW = surface seawater, OSW =overlying seawater, PW = porewater, LA = loosely attached, FA = firmly attached.

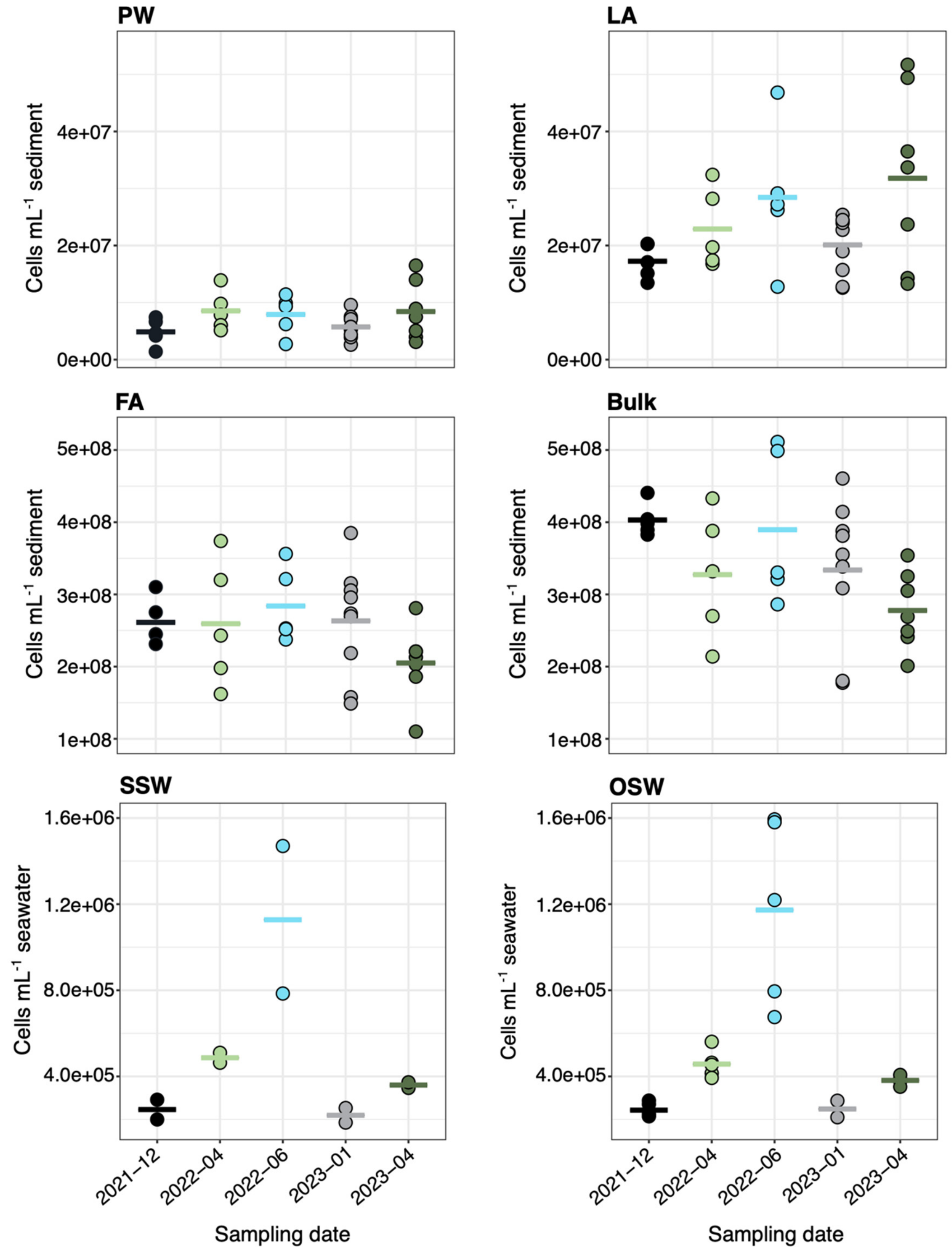

Figure S6. Cell numbers in sediment fractions, unfractionated bulk sediments, and seawater across sampling dates. Each dot represents one replicate sediment grab. The horizontal bars represent the mean. When comparing average values between polar day and polar night, cell numbers in the PW and LA cell numbers were significantly higher during the polar day (t-test: p-value = 0.011 for LA; p-value = 0.016 for PW), whereas no significant difference was observed for the FA fraction (p-value = 0.444).

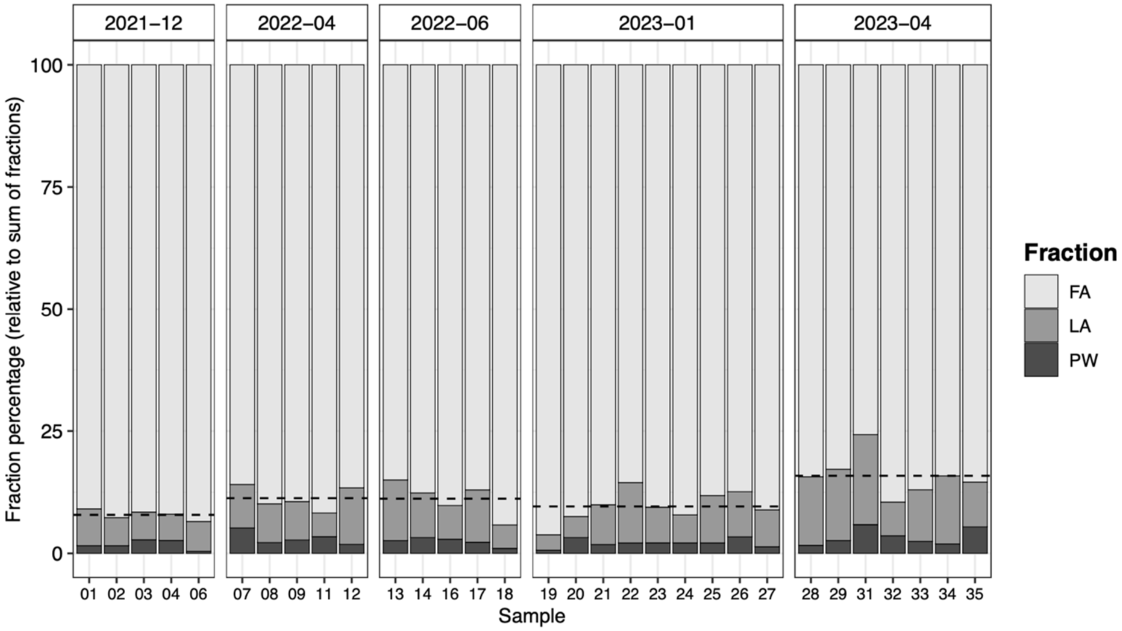

Figure S7. Cell numbers in the sediment fractions expressed as percentages relative to the sum of all fractions. Each bar represents one replicate sediment grab; x-axis labels indicate the sediment grab number. The dashed lines indicate the mean percentage of the PW and LA fractions combined. Note that cell count data from April 2022 and April 2023 have been reported previously [12].

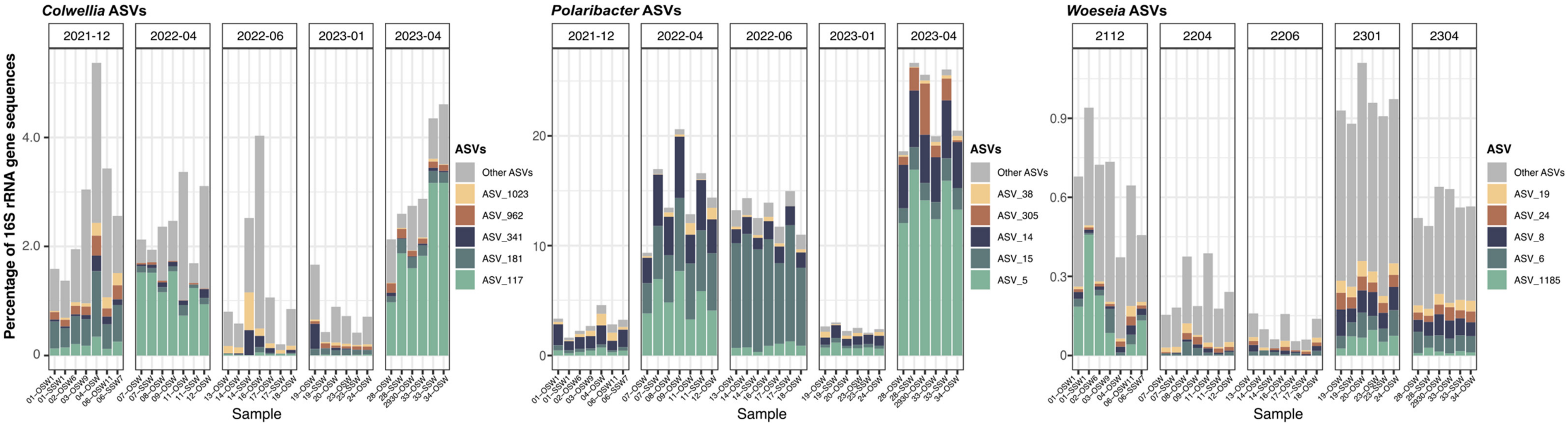

Figure S8. Changes in ASV relative abundance of selected genera in the surface seawater (SSW) and overlying seawater (OSW). The 5 most abundant ASVs in the seawater samples are shown. X-axis labels indicate the sediment grab number.

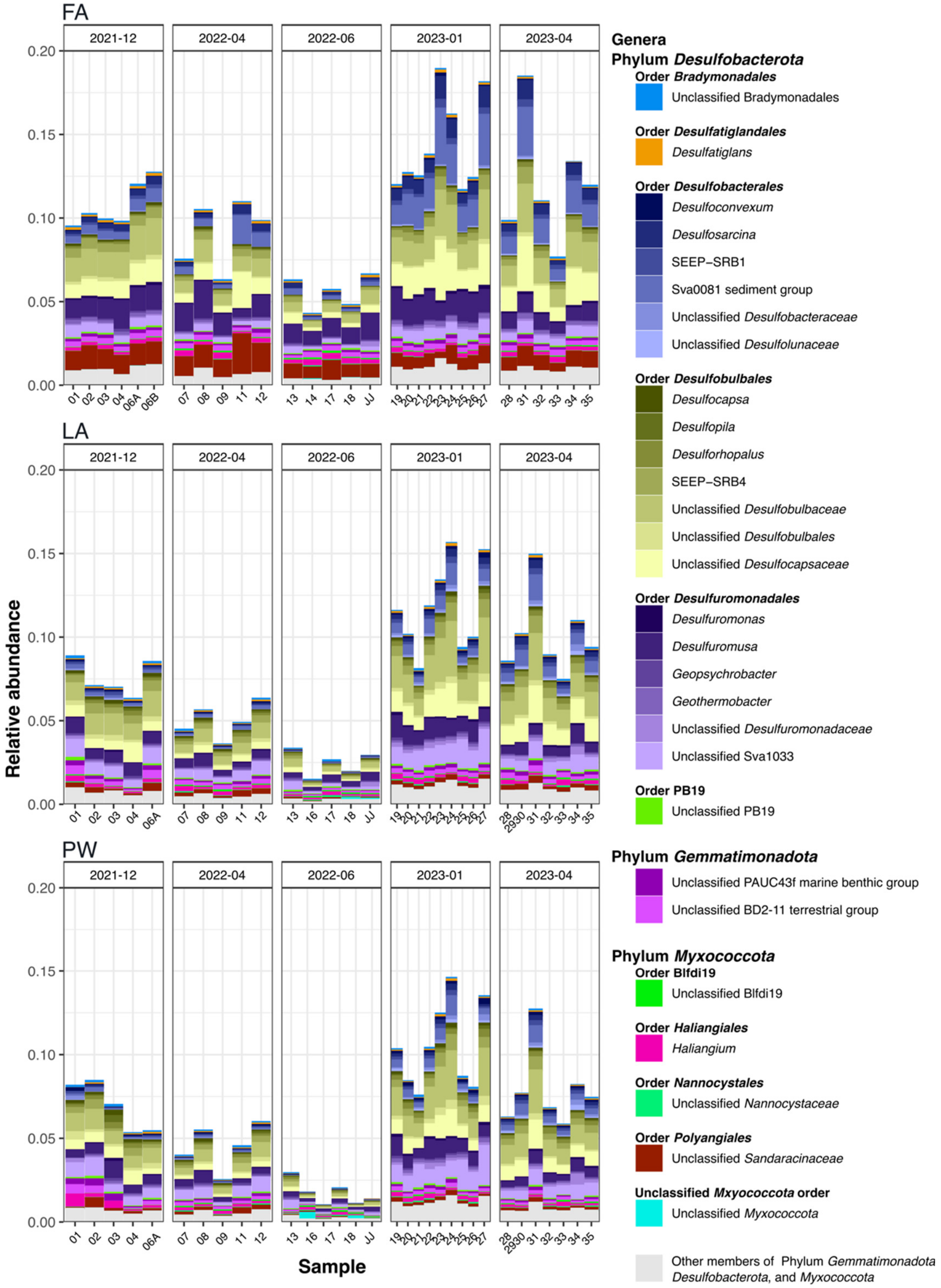

Figure S9. Seasonal changes in *Desulfobacterota*, *Myxococcota*, (formerly *Deltaproteobacteria*) and *Gemmatimonadota*. *Gemmatimonadota* was included because the DELTA495 probe also targets some members of this group. Only the 20 most abundant genera per sampling date are shown. Each bar represents one replicate sediment grab. x-axis labels indicate the sediment grab number. Genera are grouped and colored according to taxonomic order. When possible, the genus name is indicated. Otherwise, the next higher taxonomic classification is indicated.

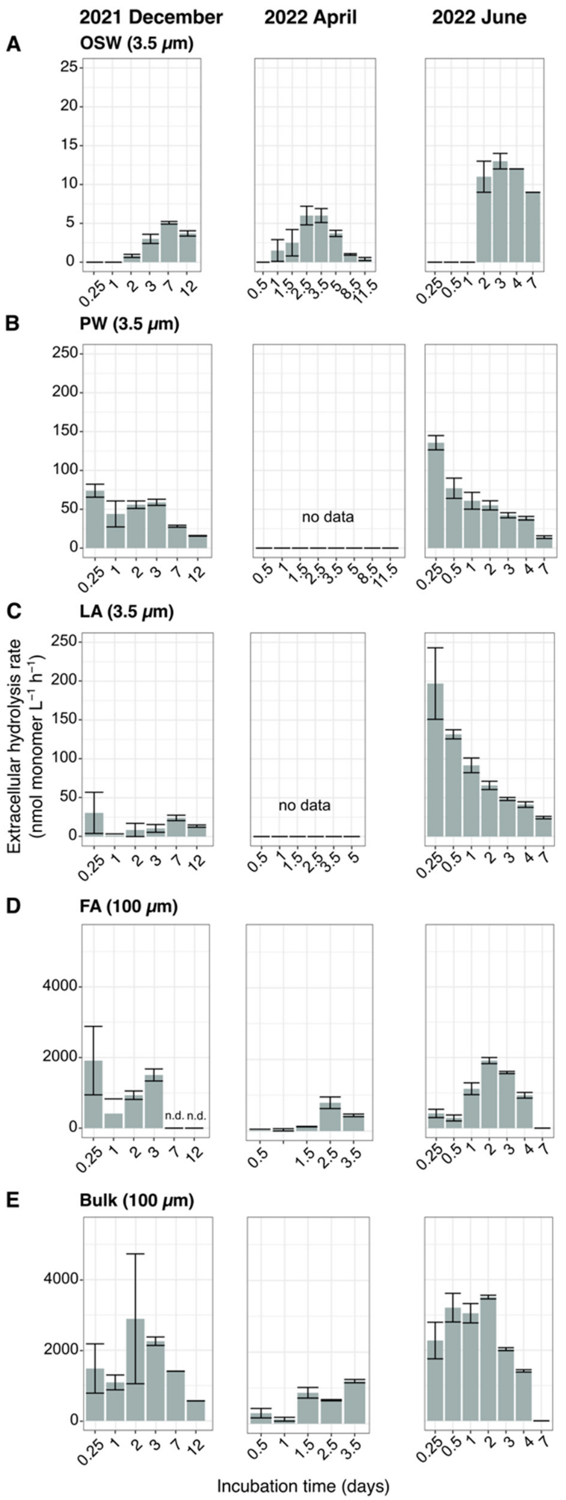

Figure S10. Mean extracellular hydrolysis rates of FLA-laminarin in the **(A)** overlying seawater, **(B, C, D)** sediment fractions, and **(E)** bulk sediment from the 2021 and 2022 incubations. The final concentrations of added FLA-laminarin are indicated in parentheses. The error bars show the standard deviation of triplicates. n.d.: no data. Note the different y-axis scales. Note that the measured rates are considered potential rates because the presence of laminarin in the environmental samples could compete with the added fluorescently-labeled polysaccharides for enzyme active sites.

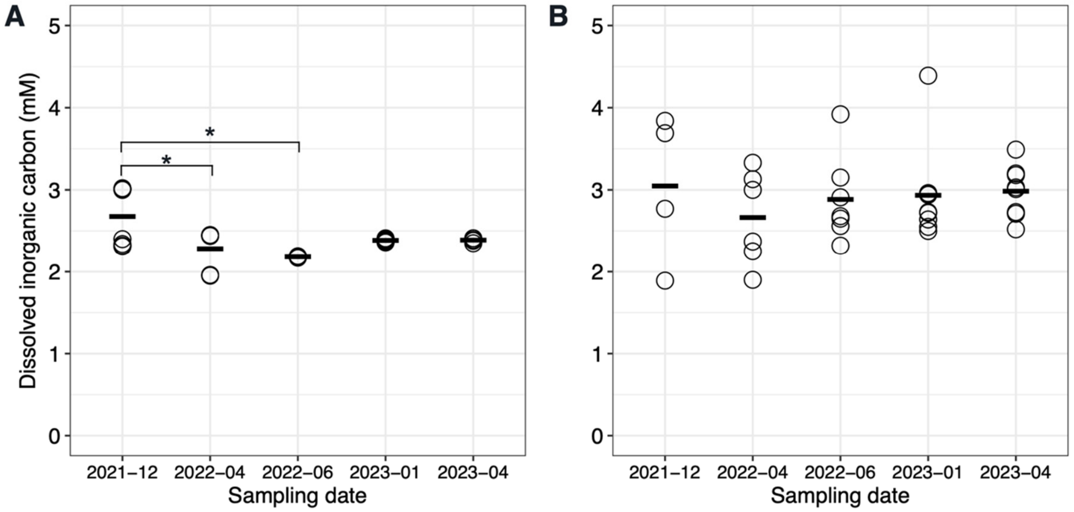

Figure S11. Dissolved inorganic carbon in **(A)** Seawater (both surface and overlying), and **(B)** porewater. Each circle represents one replicate grab. The horizontal bars represent the mean. Significance code: P-value: 0.01-0.05 ‘*’.

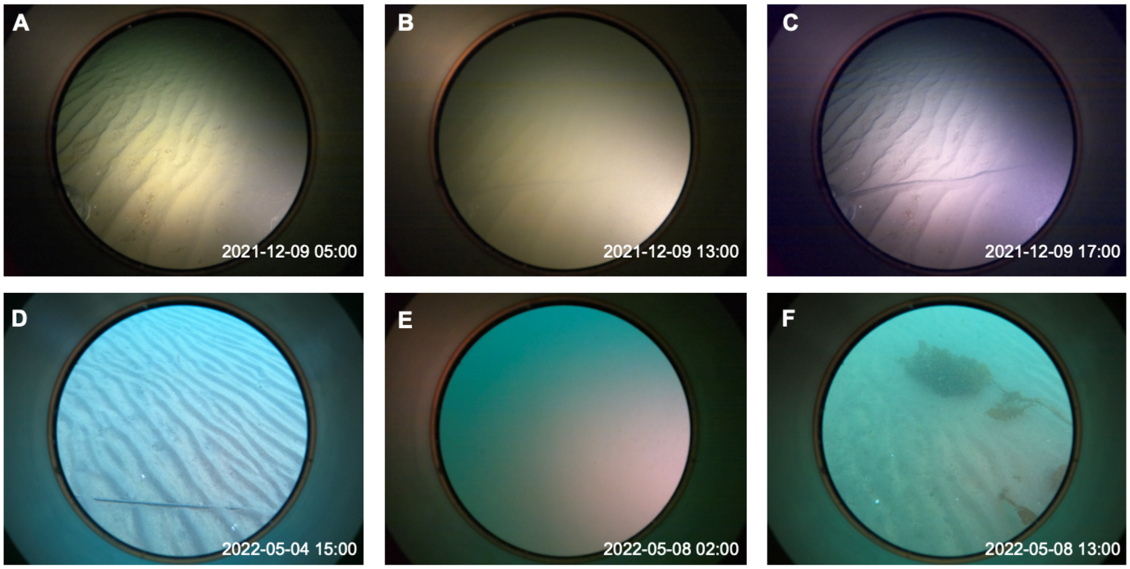

Figure S12. Images of the seafloor at Station 5, Isfjorden, Svalbard taken using an underwater camera setup. Ripples and resuspension events during polar night December 2021 **(A-C)**, and polar day May 2022 **(D-F)**.

References

1. Miksch S, Meiners M, Meyerdierks A, Probandt D, Wegener G, Titschack J, et al. Bacterial communities in temperate and polar coastal sands are seasonally stable. *ISME Commun* 2021;**1**(1):29. https://doi.org/10.1038/s43705-021-00028-w.
2. Boehnert S, Ruiz Soto S, Fox BRS, Yokoyama Y, Hebbeln D. Historic development of heavy metal contamination into the Firth of Thames, New Zealand. *Geo-Mar Lett* 2020;**40**(2):149-165. https://doi.org/10.1007/s00367-019-00597-9.
3. Meier DV, Greve AJ, Chennu A, van Erk MR, Muthukrishnan T, Abed RMM, et al. Limitation of microbial processes at saturation-level salinities in a microbial mat covering a coastal salt flat. *Appl Environ Microbiol* 2021;**87**(17):e00698-21. https://doi.org/10.1128/AEM.00698-21.
4. Hall PJ, Aller RC. Rapid, small-volume, flow injection analysis for CO2, and NH4+ in marine and freshwaters. *Limnol Oceanogr* 1992;**37**(5):1113-1119. https://doi.org/10.4319/lo.1992.37.5.1113.
5. Herlemann DPR, Labrenz M, Jürgens K, Bertilsson S, Waniek JJ, Andersson AF. Transitions in bacterial communities along the 2000 km salinity gradient of the Baltic Sea. *ISME J* 2011;**5**(10):1571-1579. https://doi.org/10.1038/ismej.2011.41.
6. Martin M. Cutadapt removes adapter sequences from high-throughput sequencing reads. *EMBnetjournal* 2011;**17**(1):3. https://doi.org/10.14806/ej.17.1.200.
7. Callahan BJ, McMurdie PJ, Rosen MJ, Han AW, Johnson AJA, Holmes SP. DADA2: High-resolution sample inference from Illumina amplicon data. *Nat Methods* 2016;**13**(7):581-583. https://doi.org/10.1038/nmeth.3869.
8. Quast C, Pruesse E, Yilmaz P, Gerken J, Schweer T, Yarza P, et al. The SILVA ribosomal RNA gene database project: improved data processing and web-based tools. *Nucleic Acids Res* 2013;**41**(D1):D590-D596. https://doi.org/10.1093/nar/gks1219.
9. R Core Team. R: A language and environment for statistical computing. R Foundation for Statistical Computing, Vienna, Austria, 2022.
10. McMurdie PJ, Holmes S. phyloseq: an R package for reproducible interactive analysis and graphics of microbiome census data. *PLoS One* 2013;**8**(4):e61217. https://doi.org/10.1371/journal.pone.0061217.
11. Oksanen J, Simpson G, Blanchet F, Kindt R, P. L, Minchin P, et al. vegan: community ecology package. R package version 2.6-4. 2022.
12. Moncada C, Arnosti C, Brüwer JD, de Beer D, Amann R, Knittel K. Niche separation in bacterial communities and activities in porewater, loosely attached, and firmly attached fractions in permeable surface sediments. *ISME J* 2024;**18**(1):wrae159. https://doi.org/10.1093/ismejo/wrae159.
